# Magnetic resonance imaging, gadolinium neutron capture therapy, and tumor cell detection using ultrasmall Gd_2_O_3_ nanoparticles coated with polyacrylic acid-rhodamine B as a multifunctional tumor theragnostic agent†

**DOI:** 10.1039/c8ra00553b

**Published:** 2018-04-03

**Authors:** Son Long Ho, Hyunsil Cha, In Taek Oh, Ki-Hye Jung, Mi Hyun Kim, Yong Jin Lee, Xu Miao, Tirusew Tegafaw, Mohammad Yaseen Ahmad, Kwon Seok Chae, Yongmin Chang, Gang Ho Lee

**Affiliations:** Department of Chemistry and Department of Nanoscience and Nanotechnology (DNN), College of Natural Sciences, Kyungpook National University (KNU) Taegu 41566 South Korea ghlee@mail.knu.ac.kr; Department of Molecular Medicine and Medical & Biological Engineering and DNN, School of Medicine, KNU and Hospital Taegu 41566 South Korea ychang@knu.ac.kr; Department of Biology Education and DNN, Teachers' College, KNU Taegu 41566 South Korea kschae@knu.ac.kr; Division of RI-Convergence Research, Korea Institute of Radiological & Medical Science Seoul 01817 South Korea

## Abstract

Monodisperse and ultrasmall gadolinium oxide (Gd_2_O_3_) nanoparticle colloids (*d*_avg_ = 1.5 nm) (nanoparticle colloid = nanoparticle coated with hydrophilic ligand) were synthesized and their performance as a multifunctional tumor theragnostic agent was investigated. The aqueous ultrasmall nanoparticle colloidal suspension was stable and non-toxic owing to hydrophilic polyacrylic acid (PAA) coating that was partly conjugated with rhodamine B (Rho) for an additional functionalization (mole ratio of PAA : Rho = 5 : 1). First, the ultrasmall nanoparticle colloids performed well as a powerful T_1_ magnetic resonance imaging (MRI) contrast agent: they exhibited a very high longitudinal water proton relaxivity (*r*_1_) of 22.6 s^−1^ mM^−1^ (*r*_2_/*r*_1_ = 1.3, *r*_2_ = transverse water proton relaxivity), which was ∼6 times higher than those of commercial Gd-chelates, and high positive contrast enhancements in T_1_ MR images in a nude mouse after intravenous administration. Second, the ultrasmall nanoparticle colloids were applied to gadolinium neutron capture therapy (GdNCT) *in vitro* and exhibited a significant U87MG tumor cell death (28.1% net value) after thermal neutron beam irradiation, which was 1.75 times higher than that obtained using commercial Gadovist. Third, the ultrasmall nanoparticle colloids exhibited stronger fluorescent intensities in tumor cells than in normal cells owing to conjugated Rho, proving their pH-sensitive fluorescent tumor cell detection ability. All these results together demonstrate that ultrasmall Gd_2_O_3_ nanoparticle colloids are the potential multifunctional tumor theragnostic agent.

## Abstract 2

Ultrasmall Gd_2_O_3_ nanoparticle colloids coated with PAA and Rho-PAA were synthesized and applied to T_1_ MRI, GdNCT and fluorescent tumor cell detection.

## Graphical abstract

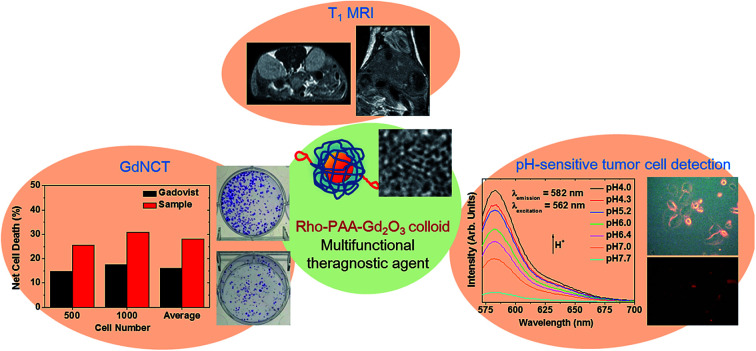

## Introduction

The superior physical properties of Gd (see Abbreviations) applicable to various biomedical applications such as T_1_ MRI,^1,2^ CT,^3,4^ and GdNCT^5–7^ provide us with the opportunity to synthesize tumor theragnostic agents using one element of Gd in various chemical forms. This condition of Gd simplifies the design and synthesis of tumor theragnostic agents, and minimizes the production cost. The effectiveness of Gd is most significant in the form of ultrasmall Gd_2_O_3_ nanoparticle colloids (nanoparticle colloid = nanoparticle coated with hydrophilic ligand) owing to their high Gd-density per nanoparticle colloid. An additional advantage of ultrasmall Gd_2_O_3_ nanoparticle colloids over molecular Gd-chelates is their large surface area available for additional functionalization through surface-conjugation. Ultrasmall Gd_2_O_3_ nanoparticle colloids also have advantages over conventional large Gd_2_O_3_ nanoparticle colloids in biomedical applications owing to their higher number density, higher colloidal stability, higher performance, and renal excretion ability.

**Fig. 1:**
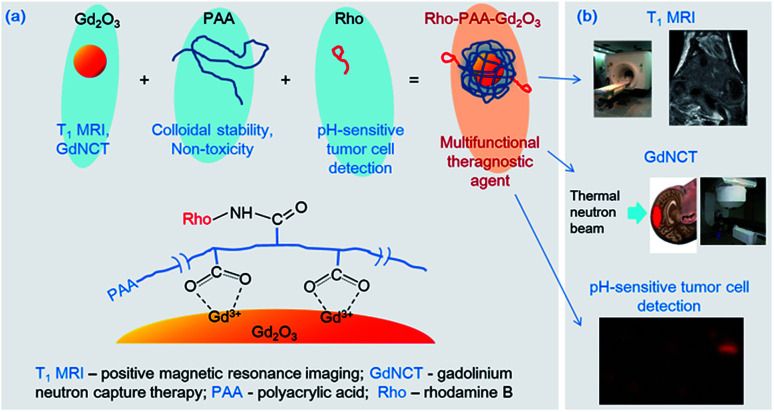
(a) Three components (*i.e.* ultrasmall Gd_2_O_3_ nanoparticle, PAA, and Rho) of the ultrasmall Gd_2_O_3_ nanoparticle colloid, the role of each component, and the surface coating structure. (b) Three applications of the ultrasmall Gd_2_O_3_ nanoparticle colloid investigated in this study.

In this study, we present monodisperse and ultrasmall Gd_2_O_3_ nanoparticle colloids as a multifunctional tumor theragnostic agent. The nanoparticle colloid is composed of three components: ultrasmall Gd_2_O_3_ nanoparticle, PAA, and Rho (Fig. 1a). First, PAA was used as surface-coating ligand to make the ultrasmall Gd_2_O_3_ nanoparticle colloids stable and non-toxic. It is an excellent surface-coating biocompatible polymer with numerous carboxylic groups per polymer,^8^ with one carboxylic group per monomer unit. An additional functionalization of the nanoparticle colloids can be easily performed by conjugating a functional molecule such as drug, tumor-targeting ligand, or dye (such as Rho in this study) to one of its carboxylic groups. The remaining carboxylic groups can be used for surface coating. The other two components are described below.

The main component, *i.e.* ultrasmall Gd_2_O_3_ nanoparticle has both diagnostic and therapeutic functions as mentioned before, and among them, those applicable to T_1_ MRI^9–11^ and GdNCT^7,12,13^ were investigated in this study. First, an important focus of this study was the application of ultrasmall Gd_2_O_3_ nanoparticle colloids to GdNCT *in vitro*. GdNCT is a new promising binary tumor therapeutic technique utilizing Gd-chemicals and a thermal neutron beam.^5,14–17^^157^Gd (natural abundance = 15.7%) has the highest thermal neutron capture cross-section of 257 000 barns among stable radionuclides, which is ∼67 times higher than 3840 barns of ^10^B (natural abundance = 19.7%) currently used for BNCT.^5,13,14^ However, common elements in the body, such as H, O, C, N, S, Na, Cl, K, Ca, and Fe, have tiny thermal neutron capture cross-section values.^5,14^ Therefore, GdNCT does no harm to our body unless high thermal neutron beam irradiation doses are used. The tumor therapy by GdNCT occurs by nuclear reaction products. Therefore, for successful GdNCT, Gd-chemicals should reside inside or close to tumor cell nuclei because of the short path lengths (a few μm) of both IC and ACK electrons, the nuclear reaction products of the ^157^Gd(n,γ)^158*^Gd nuclear reaction.^5,14^ These electrons can damage DNA inside tumor cell nuclei, thus killing tumor cells. The Gd-chemicals applied to GdNCT to date include commercial Gd-chelates such as Gd-DTPA and Gd-DOTA,^18–21^ fullerene-encapsulated Gd,^22^ GdCo@carbon nanoparticles,^23^ hybrid Gd_2_O_3_@polysiloxane nanoparticles,^12^ and various nanocomposites containing commercial Gd-chelates such as chitosans,^24,25^ liposomes,^26,27^ calcium phosphate polymeric micelles,^28,29^ and ethyl cellulose microcapsules.^30^ Nanocomposites accumulated more in tumor cells than did Gd-chelates *in vivo*^25,28,31,32^ and *in vitro*.^27,31,33,34^ However, these nanocomposites are generally too large (*d* > 30 nm) for intravenous administration. Therefore, they were usually administrated either intratumorally^24,25,32,35^ or intraperitoneally.^30,31^ However, ultrasmall nanoparticle colloids (*d* < 3 nm) can circulate freely through capillary vessels and be excreted through the renal system after intravenous administration.^9,36,37^ They may be able to target tumor cells after conjugation with targeting ligands. Therefore, ultrasmall Gd_2_O_3_ nanoparticle colloids will be extremely useful for GdNCT applications and thus were applied to GdNCT *in vitro* in this study to investigate their effectiveness as GdNCT agent before *in vivo* application. Their performance was compared to that of Gadovist (Gadobutrol, Bayer Schering Pharma, Germany), a commercial molecular T_1_ MRI contrast agent. Second, ultrasmall Gd_2_O_3_ nanoparticle colloids can be applied to T_1_ MRI. To demonstrate this, water proton relaxivities and *in vivo* T_1_ MR images in a nude mouse after intravenous administration of the sample solution were investigated in this study.

Lastly, pH-sensitive fluorescent tumor cell detection of ultrasmall Gd_2_O_3_ nanoparticle colloids was explored in this study. To this end, Rho was conjugated to surface-coating PAA through amide bond because Rho shows pH-dependent fluorescent intensity in the visible region (*λ*_em_ = 582 nm), which is useful for its use as a pH-sensitive tumor cell detection probe,^38–40^ as well as for use as an FI agent.^40,41^ Rho tints red in acidic conditions but has no color in basic conditions, and thus exhibits a stronger fluorescent intensity at more acidic conditions. The origin for this is the spirolactam ring opening reaction in acidic conditions. Therefore, ultrasmall Gd_2_O_3_ nanoparticle colloids will show stronger fluorescent intensities in tumor cells than in normal cells because the pH of tumor cells is more acidic than that of normal cells, which is nearly neutral.^42^ Therefore, combining all the applications mentioned above (Fig. 1b), the ultrasmall Gd_2_O_3_ nanoparticle colloid can serve as a multifunctional tumor theragnostic agent as demonstrated in this study.

## Experimental

### Chemicals

Chemicals such as GdCl_3_·*x*H_2_O (99.9%), PAA (*M*_w_ = ∼1800 Da, analytical standard grade), Rho (analytical standard grade), THF (>99.9%), TEG (99%), ethylenediamine (>99%), NaOH (>99.9%), dichloromethane (>99.8%), DCC (>98%), and 4-DMAP (>99%) were purchased from Sigma-Aldrich (St. Louis, MO, USA) and used as-received. Ethanol (>99%, Duksan Chemical, South Korea) was used for initial washing of nanoparticle colloids. Triple distilled water was used for final washing of nanoparticle colloids and in the preparation of aqueous nanoparticle colloidal suspension.

### Synthesis of Rho-PAA

**Fig. 2:**
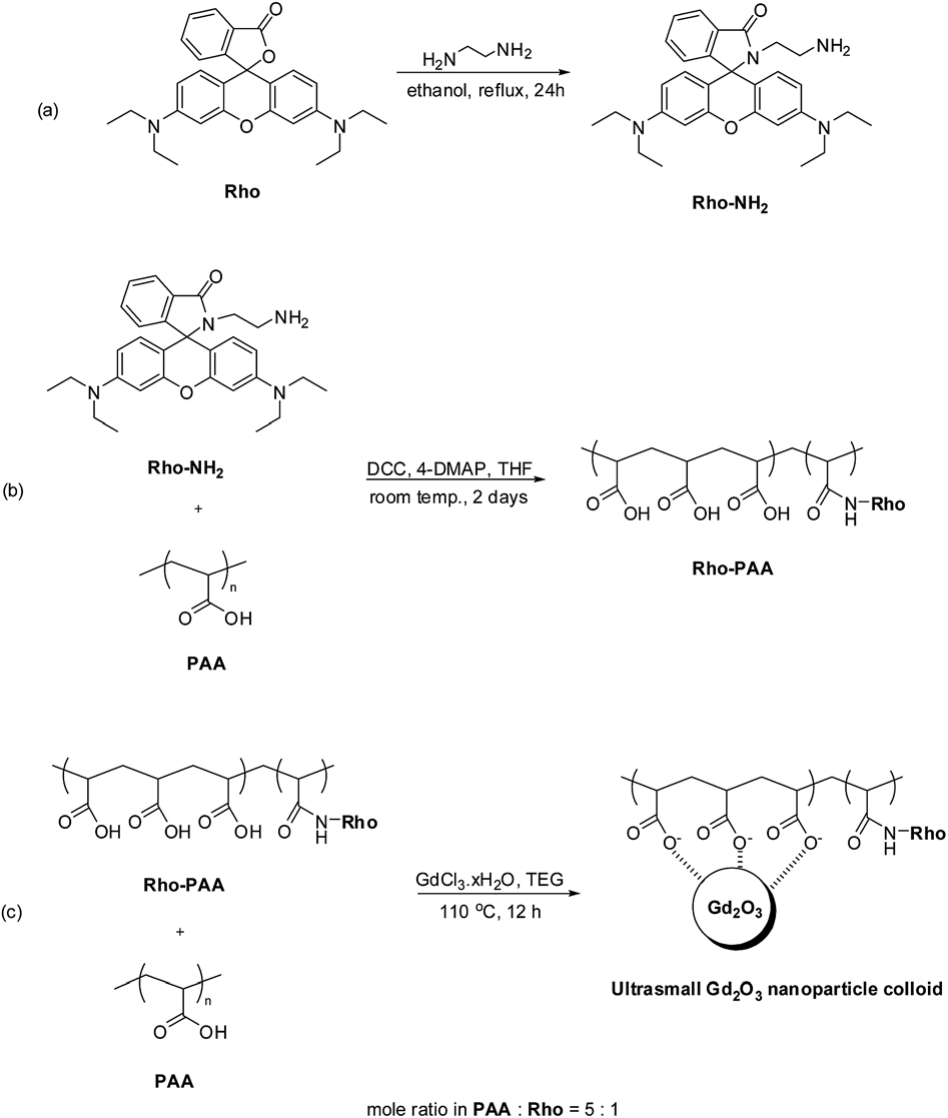
Syntheses of (a) Rho-NH_2_, (b) Rho-PAA, and (c) ultrasmall Gd_2_O_3_ nanoparticle colloid.

To prepare Rho-PAA, Rho-NH_2_ was first synthesized according to the known method (Fig. 2a).^43,44^ Briefly, a mixture of 5 mmol Rho, 20 mL ethanol, and 3 mL ethylenediamine was refluxed overnight until the reaction mixture lost its red color. Ethanol was evaporated and the crude mixture was then washed with triple distilled water, extracted with CH_2_Cl_2_, and dried by Na_2_SO_4_. The organic phase was concentrated and purified using a silica gel column (DCM : CH_3_OH = 95 : 5 as eluent) to obtain Rho-NH_2_ in a light-yellow solid form (1.86 g, 76.8% yield). Formation of Rho-NH_2_ was confirmed from its NMR and FT-IR absorption spectra (Fig. S1 and S2,† respectively).

Rho-PAA was synthesized according to the known method^45^ with a slight modification (Fig. 2b). 0.5 mmol PAA, 0.25 mmol DCC, and a catalytic amount of 4-DMAP were dissolved in 10 mL dry THF. Then 0.1 mmol Rho-NH_2_ dissolved in dry THF was slowly added to the PAA solution, which was further magnetically stirred at room temperature for 2 days. THF was later removed by evaporation and the crude product was washed with dichloromethane and purified by dialysis (MWCO = 1000 Da) against triple distilled water for 1 day. Formation of Rho-PAA was confirmed from its FT-IR absorption spectrum, and pH-dependent solution and fluorescent solution colors (Fig. S3 and S4,† respectively). The PAA used has ∼twenty-five –COOH groups per polymer. Calculations based on the mole ratio of PAA : Rho-NH_2_ = 5 : 1 used in the synthesis, showed that one of five PAA molecules was conjugated to one Rho, and the others were free PAA. Therefore, the final product was a mixture of PAA and Rho-PAA in the mole ratio of 4 : 1. This was confirmed by an FT-IR absorption spectrum of the product that was overwhelmed by PAA peaks (Fig. S3†). The product (PAA + Rho-PAA) was obtained as a solid with a red color owing to Rho-PAA. The pH-dependent solution and fluorescent solution colors were similar to those of free Rho: an aqueous solution of the product tinted red and exhibited strong fluorescence in the red region after irradiation at 365 nm with a mercury lamp, and had no color and no fluorescence at basic pH values like free Rho (Fig. S4†).

### Synthesis of ultrasmall Gd_2_O_3_ nanoparticle colloids (coating material = a mixture of PAA and PAA-Rho in the mole ratio of PAA : PAA-Rho = 4 : 1)

In a three-neck round bottom flask, a mixture of half of the above mixture (PAA + Rho-PAA) (∼0.25 mmol) and 2 mmol GdCl_3_·*x*H_2_O were magnetically stirred in 20 mL TEG for 2 h at 60 °C under atmospheric conditions until a clear red precursor solution was obtained (Fig. 2c). In a separate beaker, 10 mmol NaOH in 10 mL TEG was prepared and then added slowly to the precursor solution. Owing to the increment of pH to 9–11 after addition of the NaOH solution, the mixed solution lost its red color. The solution was magnetically stirred at 110 °C for 12 h and then cooled to room temperature. The solution was transferred to a 500 mL beaker and 400 mL ethanol was added. The solution was magnetically stirred at room temperature for 10 minutes and kept in refrigerator for 3 days until the ultrasmall Gd_2_O_3_ nanoparticle colloids settled to the bottom of the beaker. The supernatant was decanted and the remaining solution was filled with 400 mL ethanol. This process was repeated 5 times. The product solution was washed 5 times with an adequate amount (<10 mL) of triple distilled water to remove ethanol by evaporation. A portion of the product was dried in air to powder form for various characterizations and the rest of the product was diluted with triple distilled water to prepare a nanoparticle colloidal suspension (∼30 mM Gd).

### General characterizations

The particle diameters were measured using an HRTEM (Titan G2 ChemiSTEM CS Probe, FEI, Hillsboro, OR, USA) operated at an accelerating voltage of 200 kV. For measurements, one drop of diluted nanoparticle colloids in triple distilled water was dropped onto a carbon film supported by a 200-mesh copper grid (PELCO no. 160, Ted Pella, Inc., Redding, CA, USA) and placed on a filter paper using a micropipette (2–20 μL, Eppendorf, Hamburg, Germany). The copper grid with the sample was left to air dry for 1 h at room temperature. The copper grid with the sample was then mounted inside the HRTEM for measurement.

The crystal structure of the powder samples before and after TGA was measured using a powder XRD spectrometer (X-PERT PRO MRD, Philips, Eindhoven, The Netherlands) with unfiltered CuKα (*λ* = 1.54184 Å) radiation. The scanning step and scan range in 2*θ* were 0.033° and 15–100°, respectively.

The surface coating of the nanoparticles with a mixture of PAA and Rho-PAA was investigated by recording FT-IR absorption spectra with an FT-IR absorption spectrometer (Galaxy 7020A, Mattson Instruments, Inc., Madison, WI, USA). For measurements, the powder samples were dried on a hot plate at ∼40 °C for a week to remove moisture. Pellets of dried powder samples were prepared in KBr. FT-IR absorption spectra were recorded in the range of 400–4000 cm^−1^.

The amount of surface coating on the nanoparticle surface was estimated by recording a TGA curve using a TGA instrument (SDT-Q 600, TA Instruments, New Castle, DE, USA). Because organic compounds burn out below 400 °C, a TGA curve was scanned between room temperature and 900 °C under air flow. The amount of surface coating was estimated from the mass drop in the TGA curve after subtraction of the initial mass drop between room temperature and ∼105 °C owing to water and air desorption.

The Gd concentration of the nanoparticle colloids suspended in triple distilled water was determined using an ICPAES (IRIS/AP, Thermo Jarrell Ash Co., Franklin, MA, USA). The colloidal suspension was pre-treated with acids to completely dissolve the nanoparticle colloids in solution before measurement.

### *In vitro* cytotoxicity measurements

The *in vitro* cytotoxicity of the nanoparticle colloids was measured using a CellTiter-Glo Luminescent Cell Viability Assay (Promega, Madison, WI, USA). The ATP was quantified using a Victor 3 luminometer (Perkin Elmer, Waltham, MA, USA). Three cell lines, *i.e.* DU145, NCTC1469, and U87MG, were used. Each cell line was seeded onto a separate 24-well cell culture plate and incubated for 24 h (5 × 10^4^ cell density, 500 μL cells per well, 5% CO_2_, and 37 °C). Five dilute solutions were prepared by the dilution of the concentrated colloidal suspension with a sterile PBS solution. Each of the test cells was treated with ∼2 μL of each diluted sample solution and the final Gd concentrations in the treated cells were 10, 50, 100, 200, and 500 μM Gd. The treated cells were then incubated for 48 h. Cell viabilities were measured twice to obtain the average cell viabilities, which were then normalized with respect to that of untreated control cells (0.0 mM Gd).

### Relaxivity and map image measurements

The *T*_1_ and *T*_2_ relaxation times and the R_1_ and R_2_ map images were measured using a 1.5 T MRI scanner (GE 1.5 T Signa Advantage, GE Medical Systems, Chicago, IL, USA) equipped with a knee coil (MSK-Extreme, ONI Medical Systems, Inc., Wilmington, MA, USA). Five aqueous dilute sample solutions (1.0, 0.5, 0.25, 0.125, and 0.0625 mM Gd) were prepared by diluting the concentrated colloidal suspension with triple distilled water. These dilute solutions and triple distilled water were then used to measure both the *T*_1_ and *T*_2_ relaxation times and the R_1_ and R_2_ map images. The *r*_1_ and *r*_2_ water proton relaxivities of the sample solution were then estimated from the slopes of plots of 1/*T*_1_ and 1/*T*_2_, respectively, *versus* the Gd concentration. *T*_1_ relaxation time measurements were conducted using an inversion recovery method. In this method, the TI was varied at 1.5 T and the MR images were acquired at 35 different TI values in the range from 50 to 1750 ms. The *T*_1_ relaxation times were then obtained from the nonlinear least-square fits to the measured signal intensities at various TI values. For the measurements of *T*_2_ relaxation times, the Carr-Purcell–Meiboom-Gill pulse sequence was used for multiple spin-echo measurements. A total of 34 images were acquired at 34 different TE values in the range from 10 to 1900 ms. The *T*_2_ relaxation times were obtained from the nonlinear least-square fits to the mean pixel values for the multiple spin-echo measurements at various TE values.

### Animal experiment

This study was performed in accordance with the Korean guidelines and approved by the animal research committee of Kyungpook National University.

### *In vivo* T_1_ MR image measurements in a nude mouse

*In vivo* T_1_ MR images were acquired using the same MRI scanner used for relaxometry measurements. For imaging, an ICR (Institute of Cancer Research, USA) mouse (∼30 g) was anesthetized with 1.5% isoflurane in oxygen. Measurements were made before and after administration of the nanoparticle colloidal suspension into the mouse tail vein. The administration dose was typically ∼0.1 mmol Gd per kg. After measurement, the mouse was revived from anesthesia and placed in a cage with free access to food and water. During measurement, temperature of the mouse was maintained at ∼37 °C using a warm water blanket. The parameters used for the measurements were as follows: external MR field = 1.5 T; temperature = 37 °C; NEX = 4; FOV = 6 mm; phase FOV = 0.5; matrix size = 256 × 192; slice thickness = 1 mm; spacing gap = 0.5 mm (coronal) and 2.0 mm (axial); pixel bandwidth = 15.63 Hz; TR = 500 ms; and TE = 13 ms.

### *In vitro* GdNCT experiments

**Fig. 3:**
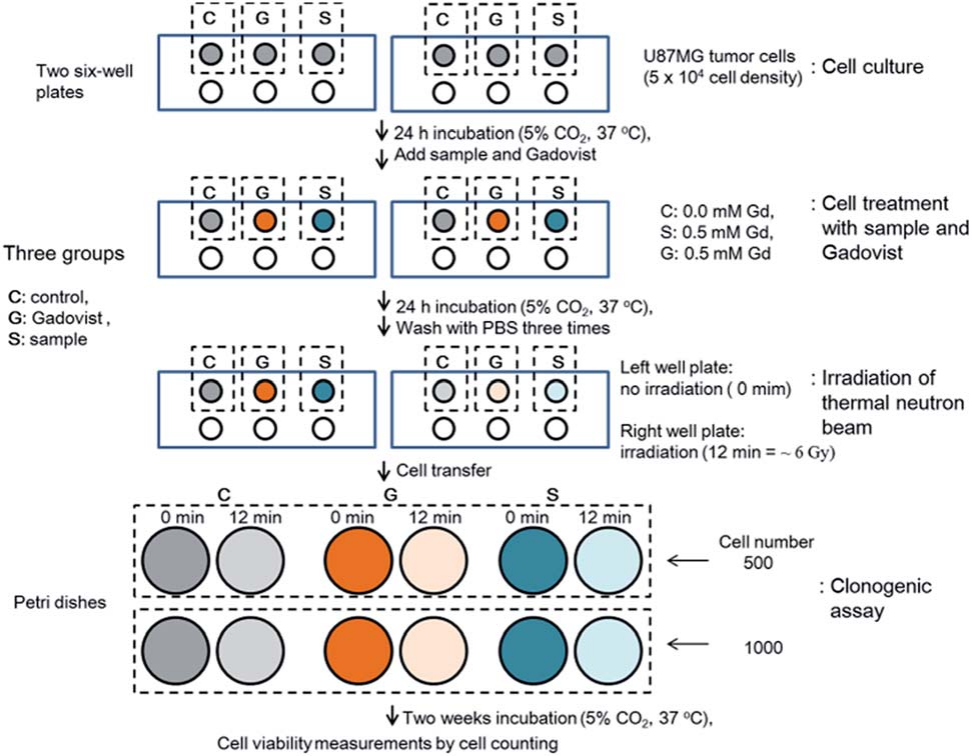
The *in vitro* GdNCT experimental procedure.

The GdNCT experiments were conducted using the cyclotron (MC50, Scanditronix, Sweden) and irradiation facilities installed at the Korea Institute of Radiological & Medical Science (Fig. S5†). It was operated at 35 MeV and 20 μA with ^9^Be target (diameter = 17 mm) to generate thermal neutron beam. The GdNCT experimental procedure is provided in Fig. 3. As shown in Fig. 3, the experiments consisted of 4 stages: cell culture, cell treatment with ultrasmall Gd_2_O_3_ nanoparticle colloids (*i.e.* sample) and Gadovist, thermal neutron beam irradiation, and clonogenic assay. Two 6-cell well plates were used for cell culture. The cell wells in each plate were divided into 3 groups (*i.e.*, control, sample, and Gadovist) such that each group occupied 1 cell well in each plate. At the cell culture stage, U87MG tumor cells were seeded on 3 cell wells in each plate and incubated for 24 h (5 × 10^4^ cell density, 5% CO_2_, and 37 °C). At the cell treatment stage, control cells were untreated (0.0 mM Gd), sample cells were treated with the sample (0.5 mM Gd), and Gadovist cells were treated with Gadovist (0.5 mM Gd). Both treated and untreated (*i.e.* control) cells were incubated for 24 h and then the treated cells were washed with PBS solution 3 times to remove free nanoparticle colloids and Gadovist. At the irradiation stage, the right cell well plate in Fig. 3 was irradiated with a thermal neutron beam for 12 min, corresponding to a radiation dose of ∼6 Gy, and the left one was not irradiated. At the clonogenic assay stage, 500 and 1000 cells in each cell well in the two 6-cell well plates were transferred to Petri dishes (diameter = 6 mm). These 2 cell numbers were used to verify consistency of cell viabilities. Twelve or 6 sets of cell dishes in total were prepared this way for clonogenic assay (see the bottom in Fig. 3). Here, each set in each group consisted of unirradiated (0 min) and irradiated (12 min) cell dishes for the 2 cell numbers. All 6 sets of cell dishes were incubated for 2 weeks to allow colonial formations. The cell viabilities were analyzed using a clonogenic assay protocol^46^ and measured by cell counting. All cells spent the same time from the cell culture to clonogenic assay.

### pH-dependent fluorescent spectral measurements

Fluorescent spectra of the ultrasmall Gd_2_O_3_ nanoparticle colloids suspended in triple distilled water at different pH values were recorded using a fluorescent spectrophotometer (Cary Eclipse, Agilent Tech., Santa Clara, CA, USA). The nanoparticle colloidal suspensions (2 mM Gd) at different pH values were prepared by addition of a small amount of 1 M HCl or 1 M NaOH solution to the sample solution while monitoring the pH value of the sample solution with a digital pH-meter equipped with a glass electrode (CyberScan pH 10, Eutech Instrument, Vernon Hills, IL, USA). For measurements, each sample solution was placed in a cuvette (Sigma-Aldrich, 4 mL) with four optically clear sides, which was put in a dark chamber of the spectrometer. A mercury lamp was used at *λ*_ex_ = 562 nm.

### Fluorescent microscopy image measurements in normal and tumor cells: pH-dependent fluorescent tumor cell detection

DU145 tumor and NCTC1469 normal cells were seeded on 24-well culture plate at a density of 1.0 × 10^4^ and 1.5 × 10^4^ per well, respectively, and incubated for 24 h in similar conditions used for cytotoxicity measurements. The cells were treated with an ultrasmall Gd_2_O_3_ nanoparticle colloidal solution (2.5 mM Gd, 2 μL), which was prepared by diluting the concentrated colloidal suspension with a sterile PBS solution, and then incubated for 24 h. After washing the treated cells with serum-free media twice, fluorescence microscopy images of the treated and untreated (*i.e.* control) cells were captured using a fluorescence microscope (IX 51, Olympus, Japan) with a mercury lamp at *λ*_ex_ = 562 nm.

## Results and discussion

### Particle diameter and crystal structure of ultrasmall Gd_2_O_3_ nanoparticle colloids

**Fig. 4:**
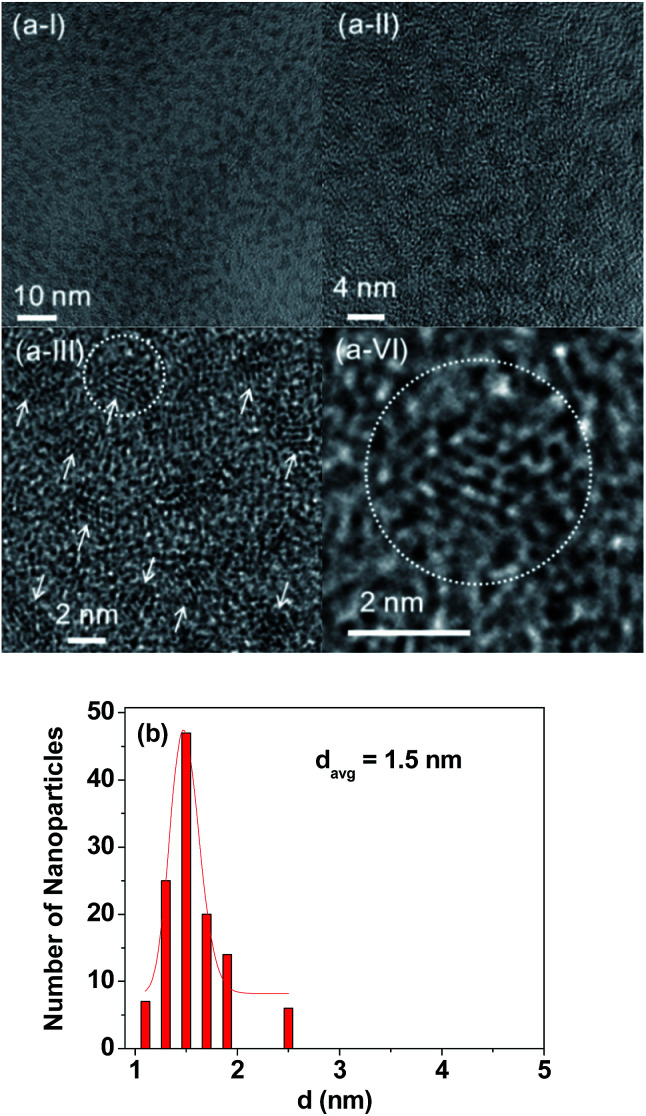
(a(I–IV)) HRTEM images at different magnifications [arrows indicate ultrasmall Gd_2_O_3_ nanoparticle colloids and the circled region in (a-III) was magnified in (a-VI)] and (b) a log-normal function fit to the observed particle diameter distribution.

As shown in HRTEM images (Fig. 4a(I–VI)), the monodisperse and ultrasmall particle diameter (*d*) ranged from 1.0 to 2.5 nm with the *d*_avg_ of 1.5 nm which was estimated from a log-normal function fit to the observed particle diameter distribution (Fig. 4b). XRD patterns before and after TGA showed that the as-prepared powder sample was amorphous owing to ultrasmall particle diameters, but the TGA-treated powder sample showed a cubic structure owing to particle growth,^47^ with a cell constant of *a* = 10.82 Å (Fig. S6 and Table S1†), which is consistent with the literature (JCPDS card no. 43-1014).^48^ In the previous experiment,^49^ we observed that free Gd^3+^ ion concentration in solution owing to dissociation of the Gd_2_O_3_ nanoparticles coated with ligands was below the detection limit of the ICPAES. Therefore, the dissociation of the Gd_2_O_3_ nanoparticle colloids will be negligible *in vitro* and *in vivo*.

### Surface coating results with a mixture of PAA and Rho-PAA

**Fig. 5:**
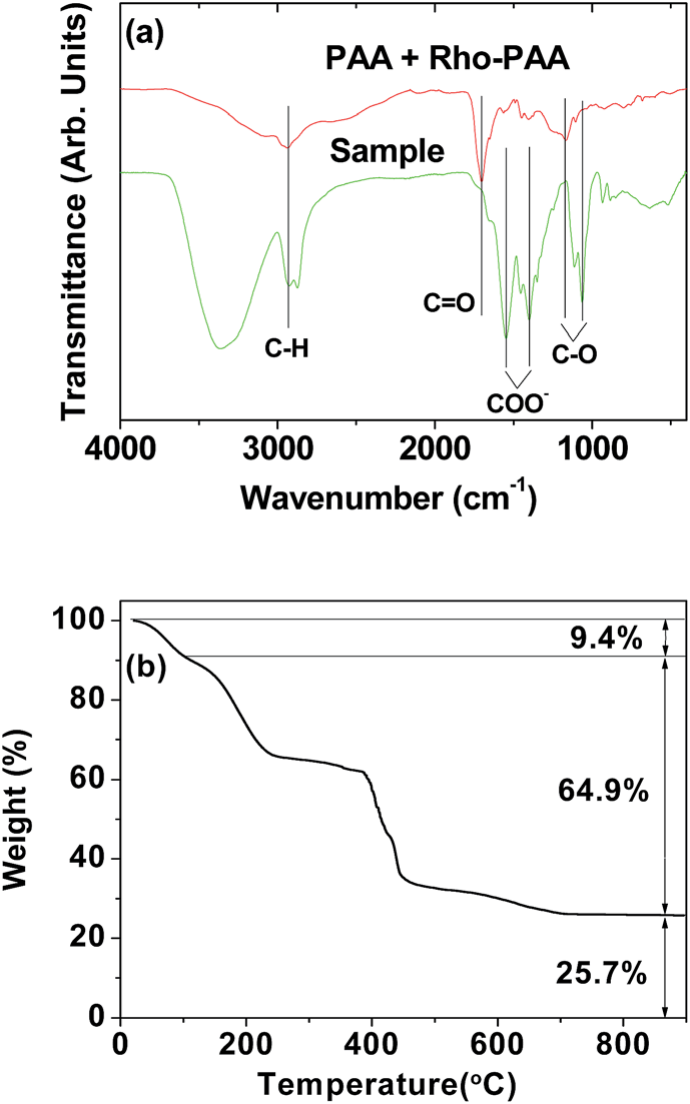
(a) FT-IR absorption spectra of a powder sample and a mixture of PAA and Rho-PAA (mole ratio of PAA : Rho = 5 : 1) 2920 cm^−1^ (C–H symmetric stretch), 1704 cm^−1^ (C=O symmetric stretch), 1550 cm^−1^ (COO^−^ antisymmetric stretch), 1400 cm^−1^ (COO^−^ symmetric stretch), and 1170 and 1065 cm^−1^ (C–O symmetric stretches). (b) A TGA curve of a powder sample.

The surface coating of a mixture of PAA and Rho-PAA on the nanoparticle surface was demonstrated by recording FT-IR absorption spectra (Fig. 5a). As shown in Fig. 5a, characteristic symmetric stretches of C–H at 2920 cm^−1^, COO^−^ at 1400 cm^−1^, and C–O at 1065 cm^−1^, and antisymmetric stretch of COO^−^ at 1550 cm^−1^ were observed in the sample, confirming the successful surface-coating of the nanoparticles with a mixture of PAA and Rho-PAA. Here, the C=O symmetric stretch of PAA and Rho-PAA at 1704 cm^−1^ was split into symmetric and antisymmetric stretches of COO^−^ in the sample and red-shifted. These are caused by electrostatic bonding between the COO^−^ of PAA and Rho-PAA, and Gd^3+^ on the nanoparticle surface (see Fig. 1a for the surface coating structure). This type of bonding corresponds to a hard acid of COO^−^ – hard base of Gd^3+^ type of interaction.^50–52^ Splitting and red-shift have been observed in many metallic oxide nanoparticles coated with ligands with –COOH groups,^53–56^ supporting our result.

The amount of surface coating with a mixture of PAA and Rho-PAA on the nanoparticle surface was estimated from a TGA curve (Fig. 5b). The initial mass drop of 9.4% between room temperature and ∼105 °C was due to water and air desorption. The mass drop of 64.9% after this was due to burning of PAA and Rho-PAA in air. The remaining mass of 25.7% was due to Gd_2_O_3_, as confirmed from its XRD pattern (Fig. S6†). Grafting density,^57^ corresponding to the average number of ligands coated per nanoparticle unit surface area, was estimated to be ∼1.5 nm^−2^ using the bulk density of Gd_2_O_3_ (7.41 g cm^−3^),^58^ the surface-coating amount of 64.9% estimated as above, and the *d*_avg_ of 1.5 nm determined by HRTEM imaging. The average number of ligands coated per nanoparticle was estimated to be ∼10 by multiplying the grafting density by the nanoparticle surface area (π*d*_avg_^2^), indicating that two Rho-PAA and eight PAA coated each nanoparticle on average.

### *In vitro* cytotoxicity results

**Fig. 6:**
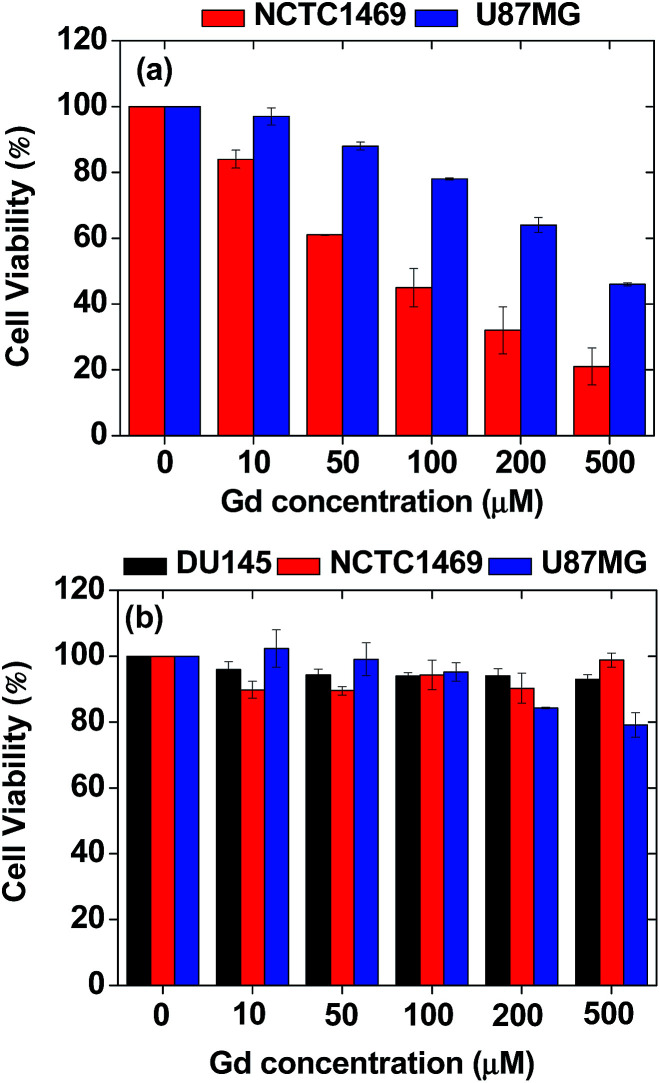
*In vitro* cytotoxicity results of (a) uncoated Gd_2_O_3_ particles in NCTC1469 and U87MG cell lines and (b) ultrasmall Gd_2_O_3_ nanoparticle colloids in DU145, NCTC1469, and U87MG cell lines.

It is well-known that gadolinium is toxic.^59^ As shown in Fig. 6a, uncoated Gd_2_O_3_ particles (purchased from Sigma-Aldrich, USA) exhibited high toxicities in both NCTC1469 normal and U87MG tumor cell lines. Gadolinium MRI contrast agents can cause nephrogenic systemic fibrosis (NSF) if free Gd^3+^ ions are deposited in tissues.^60^ Owing to this, bare Gd_2_O_3_ nanoparticles cannot be used for biomedical applications without coating. Therefore, Gd_2_O_3_ nanoparticles were coated with hydrophilic and biocompatible PAA and then functionalized with Rho in this study. As shown in Fig. 6b, the ultrasmall Gd_2_O_3_ nanoparticle colloids exhibited high cell viabilities such that ∼93% in DU145, ∼99% in NCTC1469, and ∼80% in U87MG cell lines at 500 μM Gd, showing good biocompatibility.

### Relaxivities and map images

**Fig. 7:**
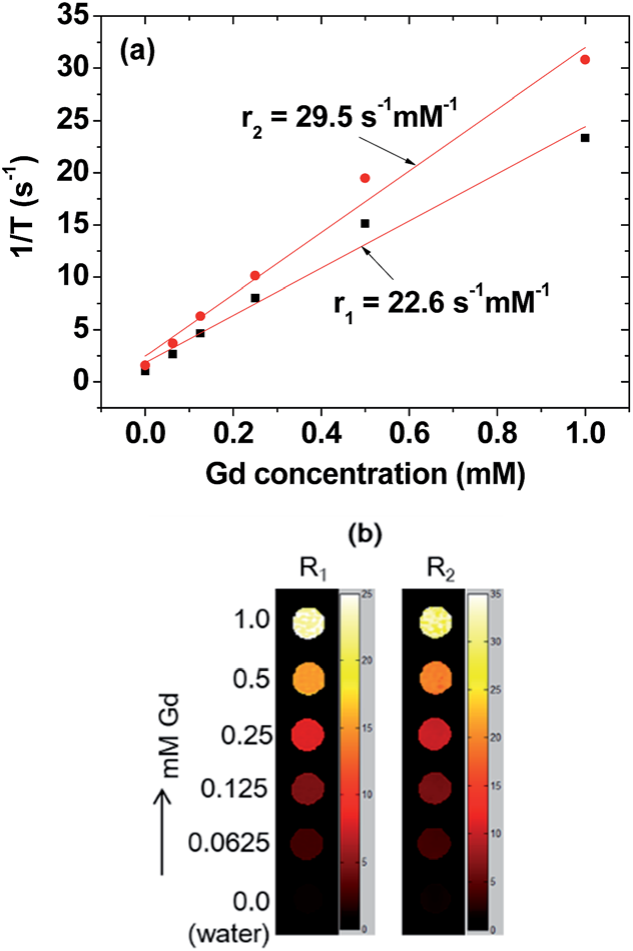
(a) Plots of 1/*T*_1_ and 1/*T*_2_ as a function of Gd concentration (the slopes correspond to *r*_1_ and *r*_2_ values, respectively). (b) Dose-dependent R_1_ and R_2_ map images of aqueous ultrasmall Gd_2_O_3_ nanoparticle colloidal suspension.

*r*_1_ and *r*_2_ values were estimated to be 22.6 and 29.5 s^−1^ mM^−1^ (*r*_2_/*r*_1_ = 1.3), respectively, from the 1/*T*_1_ and 1/*T*_2_ plots *versus* the Gd concentration (Fig. 7a). The *r*_1_ value was ∼6 times higher than those^1,2^ of commercial Gd-chelates. This higher *r*_1_ value and *r*_2_/*r*_1_ ratio (= 1.3) which is close to 1, suggested that the nanoparticle colloids would be a powerful T_1_ MRI contrast agent. This was confirmed *in vitro* from their R_1_ and R_2_ map images, clearly showing dose-dependent contrast enhancements with increasing Gd concentration (Fig. 7b). This higher *r*_1_ value is owing to ultrasmall particle size and hydrophilic surface-coating with PAA.^61^

### *In vivo* T_1_ MR images at 1.5 T

**Fig. 8:**
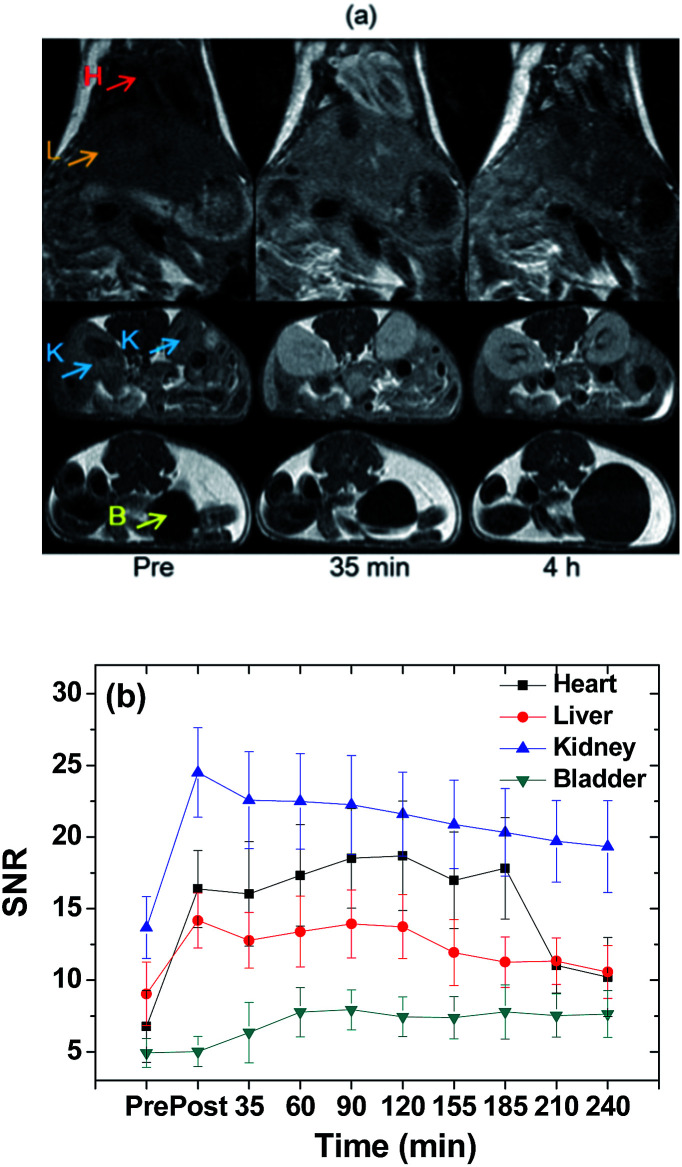
(a) *In vivo* T_1_ MR images at 1.5 T and (b) SNRs in the heart, liver, kidneys, and bladder in a nude mouse before (=pre) and 35 min and 4 h after intravenous administration. Labels: H – heart; L – liver; K – kidneys; and B – bladder.

The effectiveness of ultrasmall Gd_2_O_3_ nanoparticle colloids as a powerful T_1_ MRI contrast agent was demonstrated by recording T_1_ MR images at 1.5 T as a function of time after intravenous administration. Positive contrast enhancements were clearly observed in the liver, heart, kidneys, and bladder of a nude mouse after intravenous administration (Fig. 8a). The SNRs are plotted as a function of time (Fig. 8b). The SNRs in the liver, heart, and kidneys initially increased and then slowly decreased with time while the SNR in the bladder increased with time up to 4 h, indicating a slow renal excretion. Renal excretion is due to the ultrasmall particle size of the nanoparticle colloids, as observed by others.^9,38,62^ However, a slow renal excretion is likely related to the Rho conjugated to surface-coated PAA. The Rho may bind non-covalently to proteins in blood plasma such as albumin, fibrinogen, and globulins, as observed for other organic dyes such as FITC and fluorescein,^63^ which consequently slowed down the renal excretion. The mouse did not die after *in vivo* experiment. From the SNR plots in Fig. 8b and using the half-areas of the plots with baseline corrections after extrapolations to the pre-SNR values, the half-life of the nanoparticle colloids in the heart and liver was estimated to be ∼155 min. Therefore, the half-life of the nanoparticle colloids in the mouse would be ∼155 min.

### *In vitro* GdNCT results

**Fig. 9:**
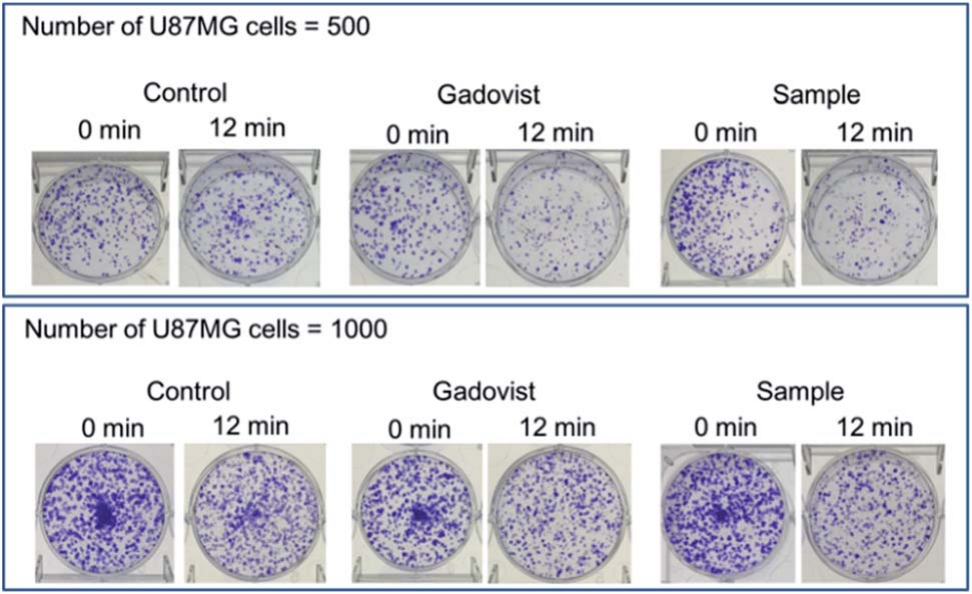
Photos of 6 sets of cell dishes containing U87MG tumor cells 2 weeks after colonial formation: control (0.0 mM Gd), Gadovist (0.5 mM Gd), and sample (0.5 mM Gd). 0 and 12 min indicate thermal neutron beam irradiation time, corresponding to 0 (*i.e.* no irradiation) and ∼6 Gy irradiation doses, respectively. All cells spent the same time from the cell culture to clonogenic assay.

**Fig. 10:**
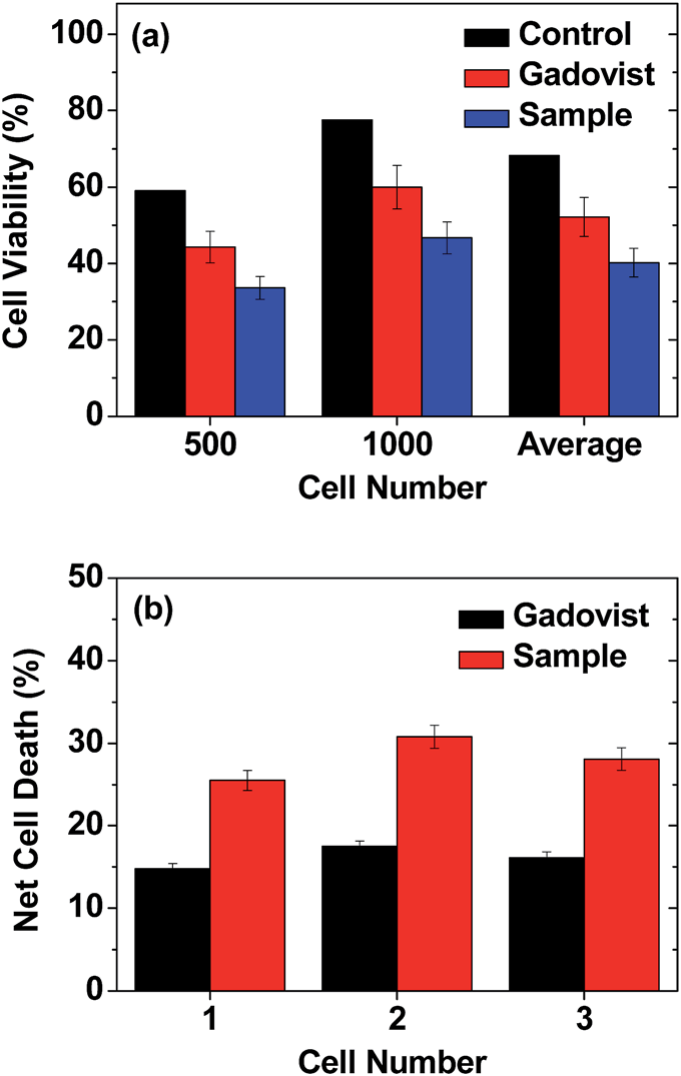
(a) Histograms of cell viabilities of irradiated U87MG tumor cells normalized with respect to those of the corresponding unirradiated cells for control, Gadovist, and sample. (b) Histograms of net cell deaths obtained by subtracting the normalized control cell death from those of sample and Gadovist.

The photos of the 6 sets of cell dishes taken 2 weeks after colonial formation are shown in Fig. 9. The cell viabilities of U87MG cells were estimated by cell counting. The cell viability of irradiated cells (12 min, Fig. 9) in each set was then normalized with respect to that of the corresponding unirradiated cells (0 min, Fig. 9). The results are plotted in histograms (Fig. 10a), showing consistency between cell viabilities of the two cell numbers. Therefore, we took an average of the cell viabilities for the two cell numbers. Cell death in the control was also observed. This was caused by the high dose of irradiation, as observed by others,^12,19,21,22,27^ which should be avoided because cells should not be damaged by thermal neutron beam itself. By subtracting the control cell death from those of sample and Gadovist, the net cell deaths of sample and Gadovist were estimated and plotted in histograms (Fig. 10b). The sample exhibited an average net cell death of 28.1%, which was 1.75 times higher than that of Gadovist. This suggests a higher cellular uptake of the ultrasmall Gd_2_O_3_ nanoparticle colloids than Gadovist, since the GdNCT effect is proportional to Gd-concentration in tumor cells as observed in various nanocomposites.^25,27,28,31,32^ This higher cellular uptake is critical for *in vivo* GdNCT experiments, and closely related to surface compositions.^64^

Gd-chemicals that have been applied to or proposed for GdNCT experiments are provided in Table 1. As given in Table 1, however, studies of GdNCT agents are not rich. Furthermore, approximately half of the studies listed in Table 1 only reported Gd concentrations accumulated in tumor cells without GdNCT experiments.^31–34,37,65–74^ Nevertheless, these previous studies demonstrated the significance and potential of GdNCT in tumor therapy: *in vitro* GdNCT studies have shown significant tumor cell death as in this study^12,19–23,27,28^ and *in vivo* GdNCT studies have shown significant tumor growth suppression.^18,21,28–30^ The limitations of the previous studies are as follows. Commercial molecular T_1_ MRI contrast agents were not suitable for *in vivo* GdNCT applications because they lack the ability to target tumor cells.^65^ Nanocomposites were generally too large to be intravenously administrated.^24,25,30–32,35^ Therefore, GdNCT agents which can circulate freely through capillary vessels after intravenous administration and then target tumor cells, are needed. In this respect, ultrasmall Gd_2_O_3_ nanoparticle colloid synthesized in this study may be one of candidates suitable for this purpose after conjugation with targeting ligand, which thus will be explored in the future.

**Table 1:** Gd-chemicals applied to or proposed for GdNCT experiments

Gd-chemical	Delivery system (particle diameter in nm)	Experimental type [*in vitro*^a^ or *in vivo* (injection type inserted)^b^]	Thermal neutron beam irradiation	Main result	Ref.
Polysiloxane-coated Gd_2_O_3_ nanoparticle	NA^c^ (3.3)	EL4-LUC cell culture	Yes	Tumor cell death	12
Na_2_[Gd(DTPA)] (dipentast)	NA (NA)	Dogs bearing oral cavity melanoma and osteosarcoma tumors (I.T.)	Yes	Comparison between BNCT and GdNCT	18
Gd-DTPA	NA (NA)	TB10 cell culture	Yes	Tumor cell death	19
Gd-DTPA	NA (NA)	SW-1573 cell culture	Yes	Tumor cell death	20
Gadobutrol (Gadovist)	NA (NA)	(i) Sk-Mel-28 cell culture; (ii) mice bearing Sk-Mel-28 tumor (I.T.)	Yes	(i) Tumor cell death; (ii) tumor growth suppression	21
Gd	C_82_ fullerene (20–30)	C-26 cell culture	Yes	Tumor cell death	22
GdCo@carbon nanoparticle	NA (20–50)	HeLa cell culture	Yes	Tumor cell death	23
Gd-DTPA	Chitosan nanoparticle (391, 214)	Mice bearing B16F10 tumor (I.T.)	Yes	Tumor growth suppression (small chitosan nanoparticle is better than large one)	24
Gd-DTPA	Chitosan nanoparticle (430)	Mice bearing B16F10 tumor (I.T.)	Yes	Tumor growth suppression better than Gd-DTPA	25
Gadoteridol (ProHance)	Liposome (100–300)	Mice bearing C-26 tumor (I.V.)	Yes	Tumor growth suppression	26
Gd-DTPA	Liposome (136–152)	F98 and LN229 cell culture	Yes	Higher tumor cell death than Gd-DTPA	27
Gd-DTPA	Calcium phosphate polymeric micelle (55)	(i) C-26 cell culture; (ii) mice bearing C-26 tumor (I.V.)	Yes	(i) Tumor cell death; (ii) tumor growth suppression better than Gd-DTPA	28
Gd-DTPA	Calcium phosphate-based nanoparticle (60)	Mice bearing C-26 tumor (I.V.)	Yes	Tumor growth suppression	29
Magnevist (gadopentetate dimeglumine)	Ethylcellulose microcapsule (75–106 μm)	Mice bearing Ehrlich ascites tumor (I.P.)	Yes	Tumor growth suppression	30
Gd(iii)	Avidin-G6-(1B4M-Gd)_254_ (∼7)	(i) SHIN3 cell culture; (ii) mice bearing SHIN3 tumor (I.P.)	No	Higher concentration of avidin-G6-(1B4M-Gd)_254_ than Gd-DTPA in tumor cell	31
Gd-DTPA	Chitosan nanoparticle (425)	Mice bearing B16F10 tumor (I.T.)	No	Higher accumulation than Gd-DTPA in tumor	32
Gd-DTPA	Chitosan nanoparticle (425)	MFH Nara-H cell culture	No	Higher accumulation than Gd-DTPA in tumor cell	33
Gd-DTPA	Chitosan nanoparticle (426)	L929, B16F10, and SCC-VII cell culture	No	Higher accumulation than Gd-DTPA in all cells	34
Gd_2_O_3_@SiO_2_@ PMPC	NA (30–200)	Mice bearing B16F10 tumor (I.T.)	No	Gd nanoparticles in tumor for 34 min by MRI	35
Gd-DTPA, Gd-DOTA	NA (NA)	(i) GBM TB10 and T98G cell culture; (ii) rats bearing C-6 tumor (I.V.) and GBM patients (I.V.)	No	(i) Gd-DTPA and Gd-DOTA found in GBM TB10 and T98G cell nuclei; (ii) Gd-DOTA found in C-6 cell nuclei and Gd-DTPA found in GBM cell nuclei	65
Gd-DTPA	Chitosan nanoparticle (3.3 μm, 4.1 μm)	—	No	Detection of γ-rays from Gd-DTPA upon thermal neutron irradiation	66
Gd-acetylacetonate	Chylomicron emulsion (100–200)	Normal nude mice (I.V.)	No	5 h circulation in blood	67
Gd-hexanedione	Microemulsion (50–125)	KB cell culture	No	Higher cellular uptake of folate-coated nanoparticles than unfolated nanoparticles	68
Gd-DTPA	Amphiphile nano assembly (3–200)	SK-MeI-28 tumor and MRC-5 normal cell culture	No	Gd compound found in both normal and tumor cells	69
Gd-DTPA	Lipid nanoemulsion (73–90)	Hamster bearing Green's melanoma (I.V.)	No	Gd in wet tumor tissue	70
Gd-DTPA	Liposome (∼100)	Mice bearing TC-1 tumor (I.V.)	No	Gd in tumor tissue	71
Gd-hexanedione	Microemulsion (85)	(i) KB cell culture; (ii) mice bearing KB tumor (I.V.)	No	Higher cellular uptake of folate-coated nanoparticles than unfolated nanoparticles	72
GdFeO_3_/Fe_3_O_4_/SiO_2_ nanoparticle	NA (∼60)	NA	No	NA	73
Gd-DTPA, Gadomer-17	PAMAM G-2 (∼3), G-4 (∼6), G-6 (∼9), G-8 (∼12), and DAB G-5 dendrimers	Normal mice (I.P.)	No	G-6 showed the highest Gd in sentinel lymph node among the seven chemicals used	74
Ultrasmall Gd_2_O_3_ nanoparticle colloid	NA (1.5)	U87MG cell culture	Yes	Net cell death = 28.1% which is 1.75 times better than Gadovist	This study

a Tumor cells used for *in vitro* GdNCT experiments: B16F10 (mouse skin melanoma); C-26 (mouse colon adenocarcinoma); EL4-LUC (mouse lymphoma); F98 (rat brain glioblastoma); KB (human nasopharyngeal epidermal carcinoma); L929 (mouse fibroblast); LN229 (human brain glioblastoma); MFH Nara-H [human sarcoma cell line malignant fibrous histiocytoma]; MRC-5 (human lung fibroblast); SCC-VII (mouse squamous cell carcinoma); SHIN3 (human ovarian cancer); Sk-Mel-28 (human melanoma); SW-1573 (human squamous lung carcinoma); TB10, T98G, and U87MG (three kinds of human GBM); TC-1 (mouse lymphocyte tumor).

b Injection type used for *in vivo* GdNCT experiments: I.V. (intravenous injection into vein); I.T. (intratumoral injection into tumor); I.P. (intraperitoneal injection into tumor inside peritoneum).

c NA: not applicable.

### pH-sensitive fluorescent tumor cell detection

**Fig. 11:**
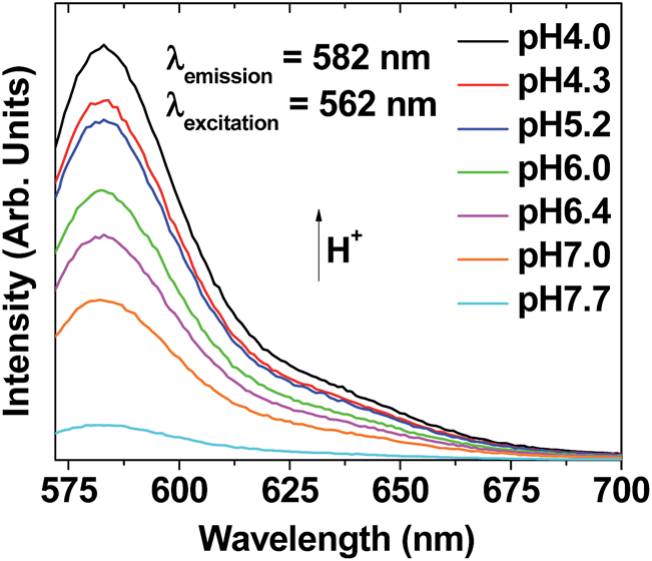
Plots of pH-dependent fluorescent spectra of aqueous ultrasmall Gd_2_O_3_ nanoparticle colloidal suspensions (2 mM Gd) (pH = 4.0–7.7) (*λ*_ex_ = 562 nm).

As expected, the aqueous ultrasmall nanoparticle colloidal suspension exhibited stronger fluorescent intensities at more acidic pH values (*λ*_em_ = 582 nm, *λ*_ex_ = 562 nm) (Fig. 11) and pH-dependent solution colors (Fig. S7†), similar to free Rho. This pH-dependent fluorescent property of the ultrasmall Gd_2_O_3_ nanoparticle colloids was applied to tumor cell detection.

**Fig. 12:**
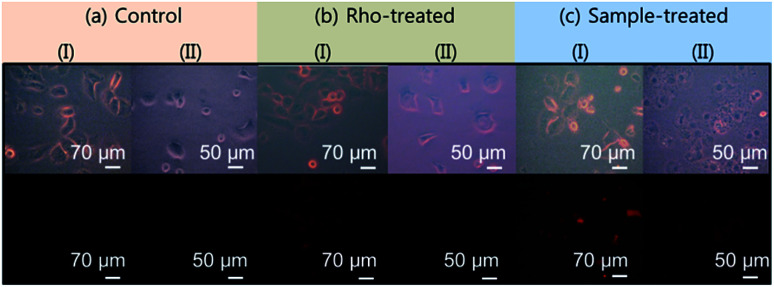
Optical (top) and fluorescent (bottom) microscopy images of (I) DU145 tumor cells and (II) NCTC1469 normal cells (a) before (=control) and after treatments with (b) Rho (1.0 mM Rho) and (c) ultrasmall Gd_2_O_3_ nanoparticle colloids (2.5 mM Gd) (*λ*_ex_ = 562 nm).

Optical and fluorescent microscopy images of both DU145 tumor and NCTC1469 normal cells were taken before and after treatments with Rho and the ultrasmall Gd_2_O_3_ nanoparticle colloids. Before treatment (=control), no fluorescence was observed in both DU145 tumor and NCTC1469 normal cells (Fig. 12a). For Rho-treated cells, a brighter fluorescence was observed in DU145 tumor cells than that in NCTC1469 normal cells, as expected because the pH of tumor cells is more acidic than that of normal cells which is nearly neutral^42^ (*λ*_ex_ = 562 nm) (Fig. 12b). This proves that Rho can serve as a pH sensitive tumor cell detection probe, as consistent with previous reports.^38–40^ For nanoparticle colloid treated cells, a stronger fluorescence was also observed in DU145 tumor cells than that in NCTC1469 normal cells owing to the conjugated Rho in the nanoparticle colloids (Fig. 12c). This result proves the pH-sensitive tumor cell detection ability of the ultrasmall nanoparticle colloids.

## Conclusions

Excellent performance of ultrasmall Gd_2_O_3_ nanoparticle colloids (*d*_avg_ = 1.5 nm) in T_1_ MRI, *in vitro* GdNCT, and pH-sensitive tumor cell detection was demonstrated. The results, as summarized below, indicate that the ultrasmall Gd_2_O_3_ nanoparticle colloids are the potential candidate for use as a multifunctional tumor theragnostic agent.

(1) Hydrophilic and biocompatible PAA was used as a surface-coating ligand that was partly conjugated with Rho through amide bond for pH-sensitive tumor cell detection (mole ratio of PAA : Rho = 5 : 1).

(2) Ultrasmall Gd_2_O_3_ nanoparticle colloids suspended in triple distilled water exhibited a very high *r*_1_ value of 22.6 s^−1^ mM^−1^ (*r*_2_/*r*_1_ = 1.3). As a result, highly positive contrast enhancements were observed in T_1_ MR images in a nude mouse after intravenous administration, proving their potential as a powerful T_1_ MRI contrast agent.

(3) Ultrasmall Gd_2_O_3_ nanoparticle colloids were applied to GdNCT *in vitro*. They showed significant U87MG tumor cell death after thermal neutron beam irradiation (0.5 mM Gd, ∼6 Gy). The net tumor cell death (corrected by tumor cell death by thermal neutron beam only) was estimated to be 28.1%, which was 1.75 times higher than that obtained using commercial Gadovist.

(4) Ultrasmall Gd_2_O_3_ nanoparticle colloids showed stronger fluorescent intensities in DU145 tumor cells than in NCTC1469 normal cells owing to conjugated Rho, proving their pH-sensitive fluorescent tumor cell detection ability.

## Author contributions

SLH synthesized and characterized the ultrasmall Gd_2_O_3_ nanoparticle colloids, XM, TT and AMY contributed to experiments, HC and YC took and analyzed MRI data, ITO and KSC measured and analyzed cytotoxicity and fluorescent microscopy image data, KHJ, MHK and YJL performed GdNCT experiments, and GHL wrote the manuscript.

## Conflicts of interest

There are no conflicts to declare.

## Abbreviations

ACK: Auger–Coster–Kronig
ATP: Intracellular adenosine triphosphate
1B4M: 2-(*p*-Isothiocyanatobenzyl)-6-methyl-diethylenetriaminepentaacetic acid
BNCT: Boron neutron capture therapy
C: Carbon
Ca: Calcium
Cl: Chlorine
CT: X-ray computed tomography
Da: Dalton
DAB: Polypropylenimine dendrimer
*d*_avg_
: Average particle diameter
DCC: *N*,*N*′-Dicyclohexylcarbodiimide
4-DMAP: 4-(Dimethylamino)pyridine
DOTA: 1,4,7,10-Tetraazacyclododecane-1,4,7,10-tetraacetic acid
DTPA: Diethylenetriaminepentaacetic acid
DU145: Human prostate cancer
Fe: Iron
FI: Fluorescent imaging
FITC: Fluorescein-isothiocyanate
FOV: Field of view
FT-IR: Fourier transform-infrared
G6: Generation-6 polyamidoamine dendrimer
GBM: Glioblastoma multiforme
Gd: Gadolinium
GdNCT: Gadolinium neutron capture therapy
Gd_2_O_3_: Gadolinium oxide
H: Gydrogen
HRTEM: High-resolution transmission electron microscope
IC: Internal conversion
ICPAES: Inductively coupled plasma atomic emission spectrometer
ICR: Institute for Cancer Research
K: Potassium
*λ*_em_
: Emission wavelength
*λ*_ex_
: Excitation wavelength
*M*_w_
: Molecular weight
MWCO: Molecular weight cut off
MR: Magnetic resonance
N: Nitrogen
Na: Sodium
NCTC1469: Normal mouse hepatocyte
NEX: Number of acquisition
NMR: Nuclear magnetic resonance
O: Oxygen
PAA: Polyacrylic acid
PAMAM: Polyamidoamine
PBS: Phosphate-buffered saline
PMPC: Poly(2-methacryloyloxyethyl phosphorylcholine)
R_1_: Longitudinal relaxation map
*r*_1_
: Longitudinal water proton relaxivity
R_2_: Transverse relaxation map
*r*_2_
: Transverse water proton relaxivity
Rho: Rhodamine B
S: Sulfur
SNR: Signal to noise ratio
T: Tesla
*T*_1_
: Longitudinal relaxation time
*T*_2_
: Transverse relaxation time
TE: Echo time
TEG: Triethylene glycol
TGA: Thermogravimetric analysis
THF: Tetrahydrofuran
TI: Tnversion time
T_1_ MRI: Positive magnetic resonance imaging
TR: Repetition time
U87MG: Human malignant glioblastoma tumor cell line
XRD: X-ray diffraction

## Supplementary Material

RA-008-C8RA00553B-s001
