# Supplementary material for: Magnetic resonance imaging, gadolinium neutron capture therapy, and tumor cell detection using ultrasmall Gd2O3 nanoparticles coated with polyacrylic acid-rhodamine B as a multifunctional tumor theragnostic agent
Source: RSC Adv. 2018 Apr 3;8(23):12653–65. doi: 10.1039/c8ra00553b (PMC9079332; doi:10.1039/c8ra00553b)
Supplement: RA-008-C8RA00553B-s001 [file RA-008-C8RA00553B-s001.pdf]

Electronic Supplementary Material (ESI) for RSC Advances.
This journal is © The Royal Society of Chemistry 2018

Supplementary Information

Title: Magnetic resonance imaging, gadolinium neutron capture therapy, and tumor cell detection using ultrasmall Gd_2O_3 nanoparticles coated with polyacrylic acid-rhodamine B as a multifunctional tumor theragnostic agent

Authors: Son Long Ho, Hyunsil Cha, In Taek Oh, Ki-Hye Jung, Mi Hyun Kim, Yong Jin Lee, Xu Miao, Tirusew Tegafaw, Mohammad Yaseen Ahmad, Kwon Seok Chae, Yongmin Chang and Gang Ho Lee

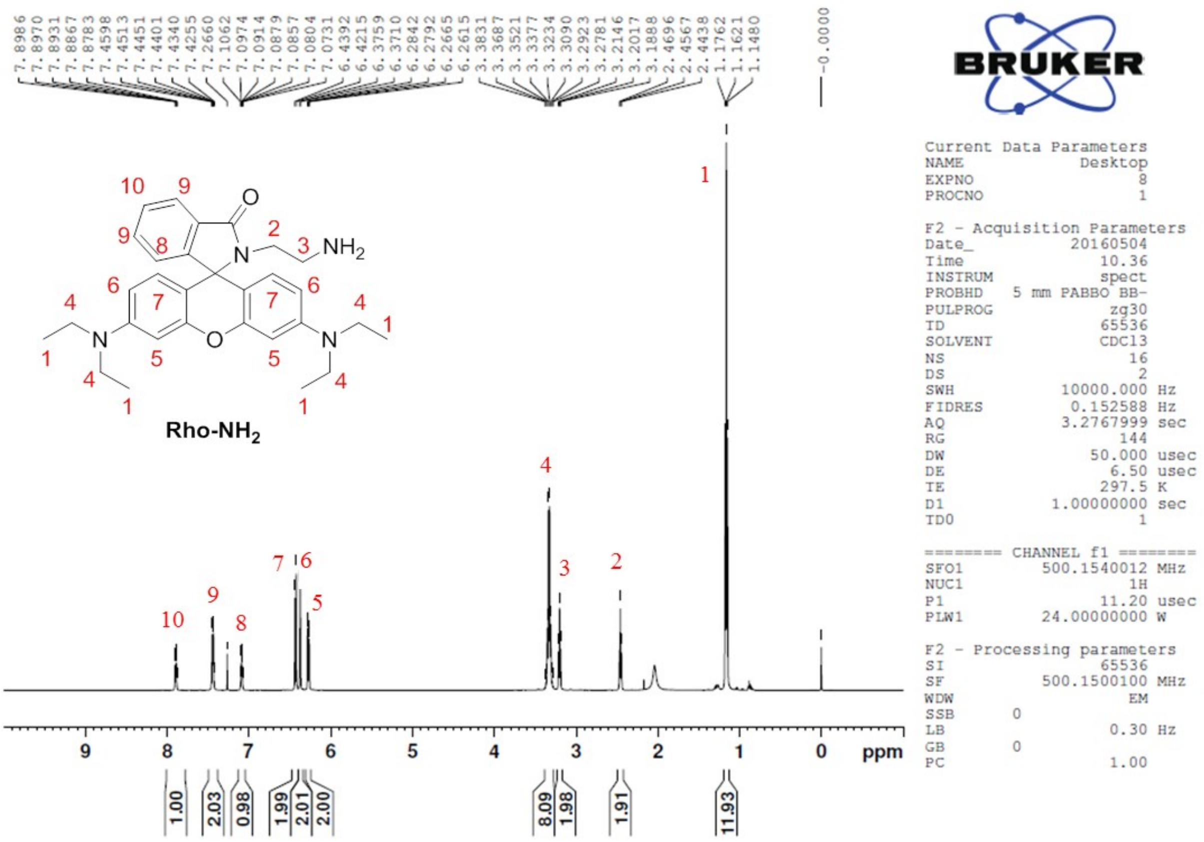

Fig. S1 An NMR spectrum of Rho-NH_2.

(1) NMR and FT-IR absorption spectra of Rho-NH_2

A successful synthesis of Rho-NH_2 was confirmed from its NMR and FT-IR absorption spectra. In the NMR spectrum, all peaks could be assigned (Fig. S1). All characteristic absorption peaks of Rho appeared in the FT-IR absorption spectrum of Rho-NH_2 with an additional N-H stretching vibration at 3370 cm^{-1} (Fig. S2). The C=O stretch of Rho at 1749 cm^{-1} was red-shifted to 1682 cm^{-1} in Rho-NH_2, owing to amide bond formation in which the electron attracting nitrogen weakened the C=O bond. The benzene ring C=C stretches[1,2] in both Rho and Rho-NH_2 were observed at 1610 and 1510 cm^{-1}. This additionally supported the formation of Rho-NH_2.

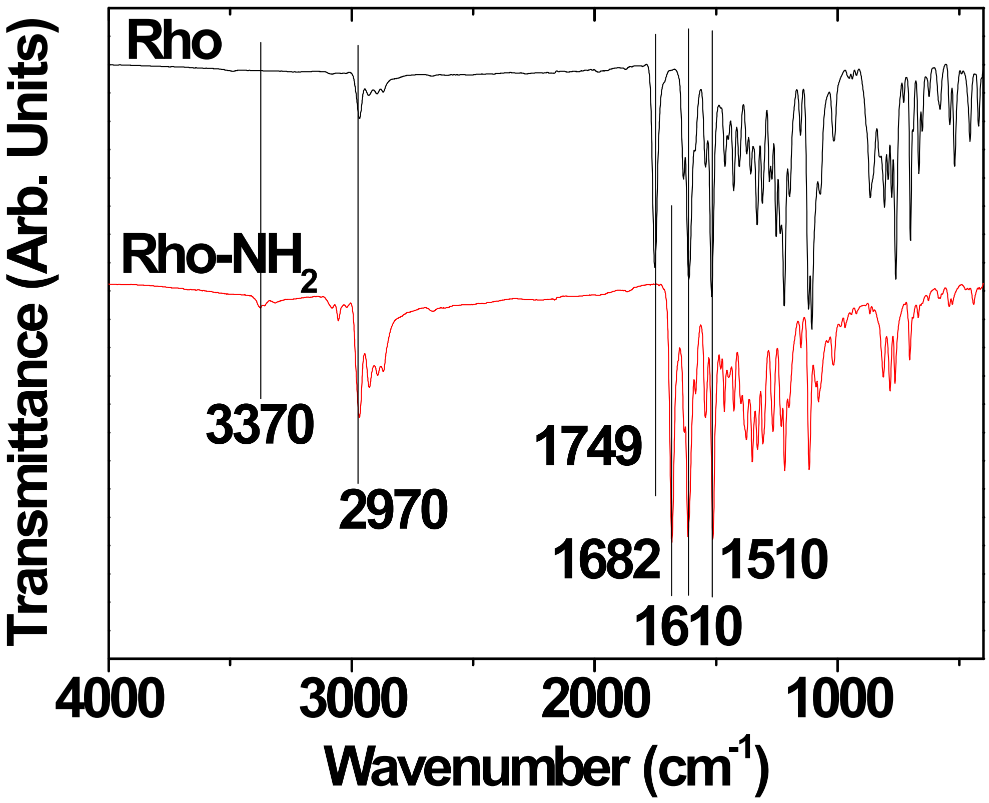

Fig. S2 FT-IR absorption spectra of Rho (top) and Rho-NH_2 (bottom): 3370 cm^{-1} (N-H symmetric stretch), 2970 cm^{-1} (C-H symmetric stretch), 1749 and 1682 cm^{-1} (C=O symmetric stretches), and 1610 and 1510 cm^{-1} (benzene ring C=C stretches).

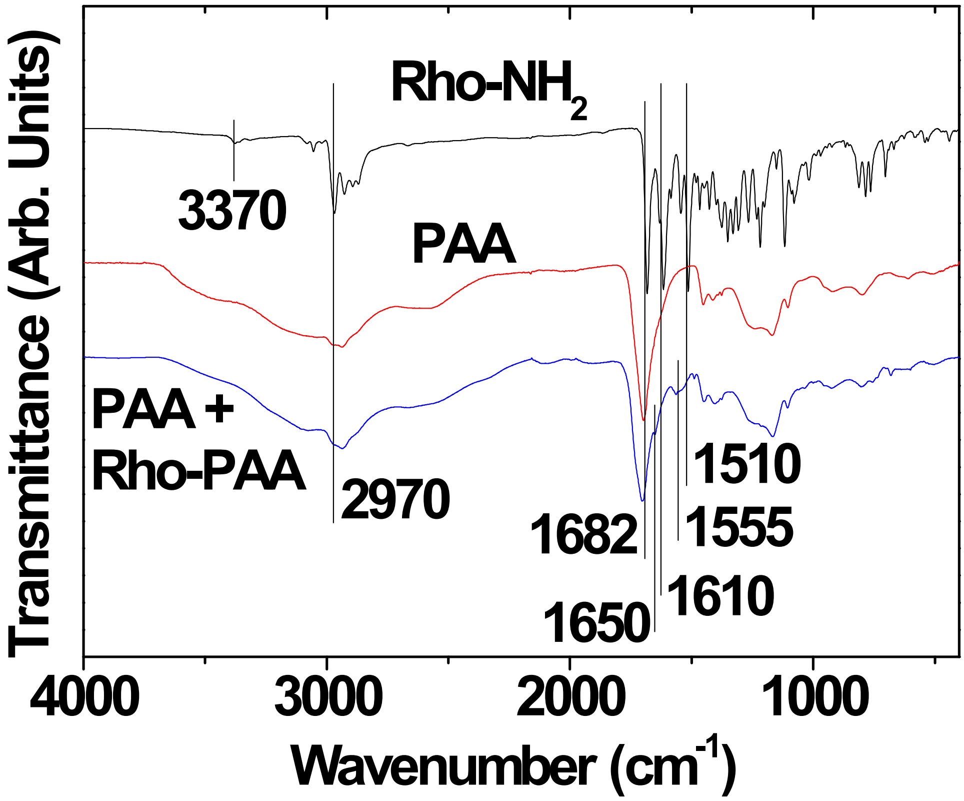

Fig. S3 FT-IR absorption spectra of Rho-NH_2, PAA, and a mixture of PAA and Rho-PAA (mole ratio of PAA : Rho = 5 : 1): 3370 cm^{-1} (N-H symmetric stretch), 2970 cm^{-1} (C-H symmetric stretch), 1682 cm^{-1} (C=O symmetric stretch), and 1650, 1610, 1555, and 1510 cm^{-1} (benzene ring C=C stretches).

(2) An FT-IR absorption spectrum and pH-dependent solution colors of Rho-PAA with and without UV irradiation

The mole ratio of PAA : Rho used was 5 : 1. Therefore, one-fifth of PAA was in the form of PAA-Rho and the others were free PAA, and each PAA-Rho contained one Rho. Therefore, the FT-IR absorption spectrum of a mixture of PAA and Rho-PAA was similar to that of PAA (Fig. S3). The small peaks at 1650 and 1555 cm^{-1} in the FT-IR spectrum of a mixture of PAA and Rho-PAA were

owing to benzene ring C=C stretches of Rho-PAA, and slightly blue-shifted from 1610 and 1510 cm^{-1} of free Rho-NH_2 or free Rho, respectively. These two peaks confirmed the formation of Rho-PAA. Another evidence for the formation of Rho-PAA was the pH-dependent solution and fluorescent solution colors. Like free Rho, an aqueous solution of a mixture of PAA and Rho-PAA revealed pH-dependent solution colors (Fig. S4a) and fluorescent solution colors (Fig. S4b). The solution tinted red at acidic pH values but had no color (or transparent) at basic pH values after addition of 1.0 M NaOH solution, similar to free Rho (Fig. S4a). After irradiation at λ_{ex} = 365 nm with a mercury lamp, the solution tinted red at an acidic pH value but had no color at a basic pH value like free Rho (Fig. S4b).

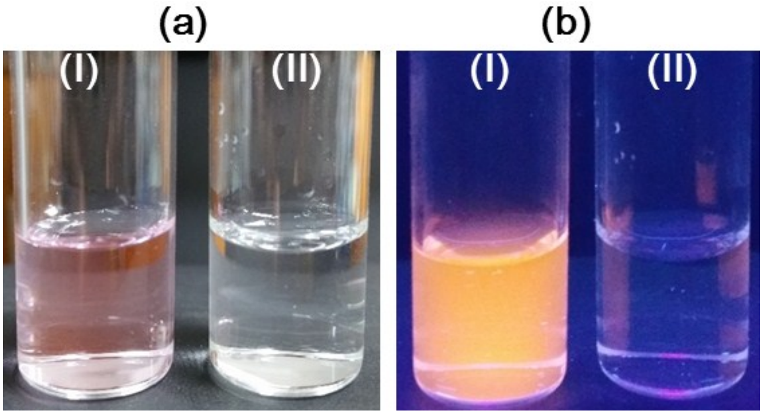

Fig. S4 (a) Aqueous solution colors of a mixture of PAA and Rho-PAA (total 0.1g/L) and (b) solution colors after irradiation at λ_{ex} = 365 nm with a mercury lamp: (I) acidic and (II) basic pH conditions.

(3) GdNCT experimental facilities

The GdNCT experiments were conducted using the cyclotron (MC50, Scanditronix, Sweden) and beam irradiation facilities installed at the Korea Institute of Radiological & Medical Science (Fig. S5). The cyclotron was operated at 35 MeV and 20 μA with ^{9}Be target (diameter = 17 mm) to generate thermal neutron beam.

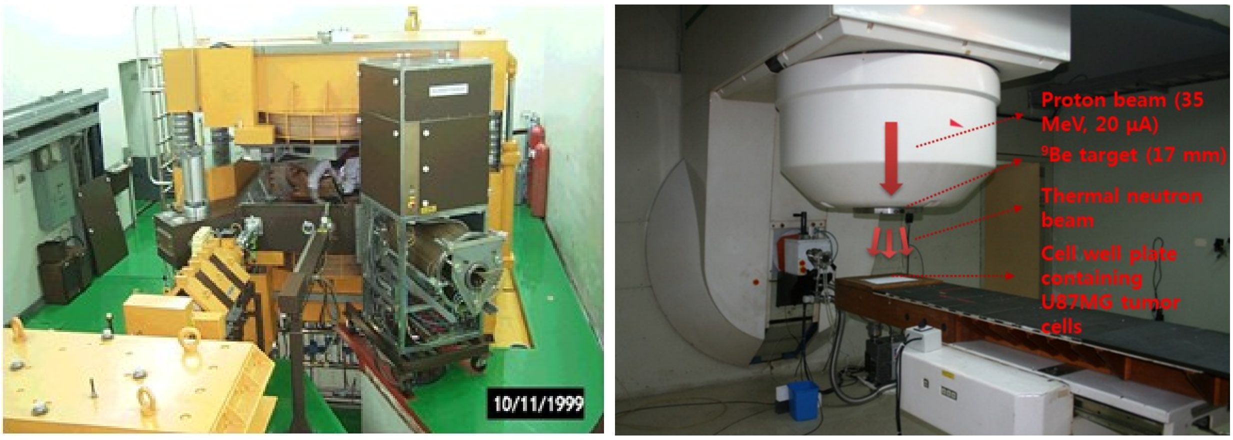

Fig. S5 The MC50 cyclotron (left) and thermal neutron beam irradiation (right) facilities at the Korea Institute of Radiological & Medical Science.

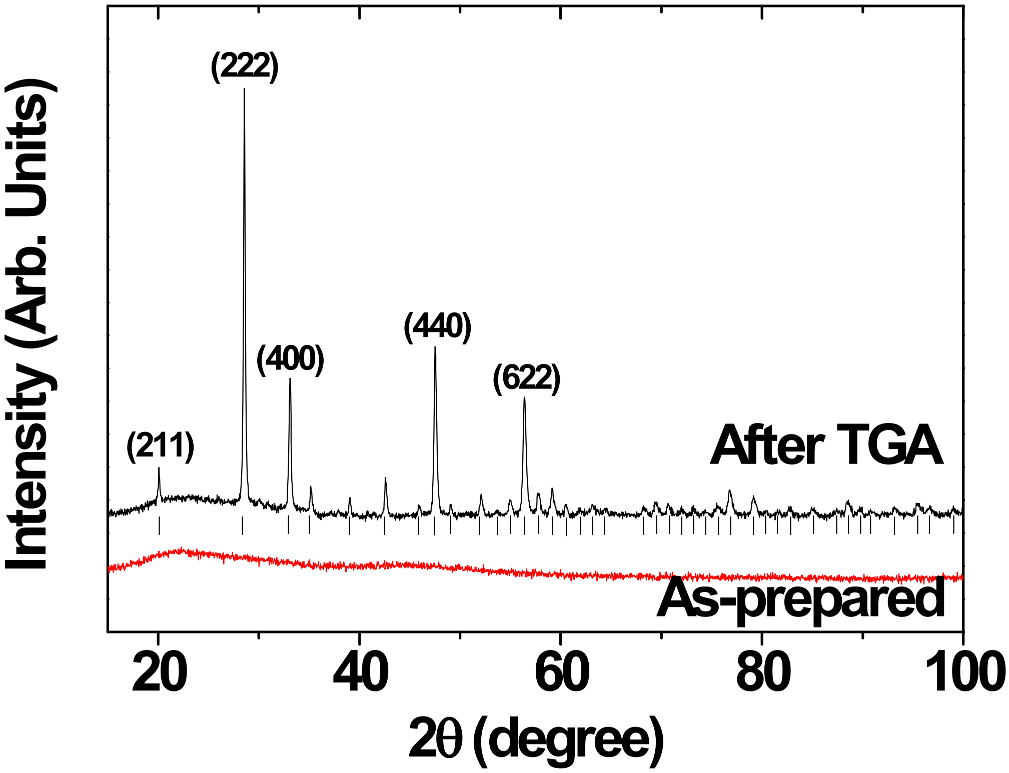

Fig. S6 XRD patterns of as-prepared (bottom) and TGA-treated (top) powder samples. The assignments at the top XRD pattern are the (hkl) Miller indices. All the observed peaks (labelled as vertical bars) are assigned in Table S1.

(4) XRD patterns before and after TGA

The XRD pattern of the powder sample of as-prepared ultrasmall Gd_2O_3 nanoparticle colloids was amorphous (the bottom XRD pattern in Fig. S6), owing to ultrasmall particle diameters, whereas a cubic structure with a cell constant of a = 10.82 Å was observed after TGA owing to particle growth (the top XRD pattern in Fig. S6). The estimated cell constant of TGA-treated sample was consistent with the literature (JCPDS card No. 43-1014).[3] Only strong peaks were assigned with (hkl) Miller indices in Fig. S6. Assignments of all the observed peaks (labelled as vertical bars) are provided in Table S1.

Table S1 Assignment of all the observed peaks in the XRD pattern of the TGA-treated sample

(hkl)	2θ	(hkl)	2θ	(hkl)	2θ
211	20.120	444	59.164	833	80.341
222	28.569	543	60.554	842	81.530
400	33.141	046	61.852	655	82.718
411	35.171	633	63.196	158	85.004
332	38.993	642	64.431	763	87.382
134	42.577	156	68.308	844	88.580
125	45.961	800	69.496	853	89.777
440	47.533	811	70.749	860	90.870
433	49.124	820	71.984	268	93.206
611	52.105	653	73.172	1022	95.530
026	53.696	822	74.379	765	96.630
145	54.995	831	75.568	871	99.010
622	56.384	662	76.760	-	-
631	57.774	840	79.134	-	-

(5) pH-dependent sample solution colors with and without UV irradiation

Like free Rho,[4-6] ultrasmall Gd_2O_3 nanoparticle colloidal suspension tinted red at acidic conditions

but had no color at basic conditions (Fig. S7a). Its fluorescent solution color tinted red at low pH values like free Rho, but blue at pH values ≥ 6.0 unlike free Rho (λ_{ex} = 365 nm) (Fig. S7b). This unexpected blue color is simply due to a strong 365 nm blue-light scattering by the ultrasmall nanoparticle colloids (so called the Tyndall effect), which overwhelmed the solution color: i.e. no color was expected like an aqueous solution of a mixture of PAA and Rho-PAA as given in Fig. S4 or free Rho.

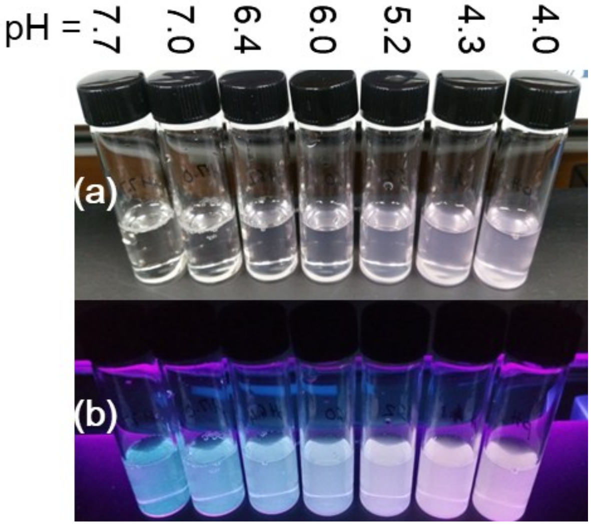

Fig. S7 Photos of the nanoparticle colloidal suspensions (a) before and (b) after irradiation with λ_{ex} = 365 nm.

References

1 T. M. Geng, R. Y. Huang and D. Y. Wu, *RSC Adv*., 2014, **4**, 46332-46339.

2 N. O. Mchedlov-Petrosyan, L. A. Fedorov, S. A. Sokolovskii, Y. N. Surov and R. S. Maiorga, *Russ. Chem. Bull*., 1992, **41**, 403-409.

3 a = 10.813 Å. JCPDS-International Centre for Diffraction Data, card no. 43-1014, PCPDFWIN, vol. 1.30, 1997.

4 S.-L. Shen, X.-P. Chen, X.-F. Zhang, J.-Y. Miao and B.-X. Zhao, *J. Mat. Chem. B*, 2015, **3**, 919-925.

5 Q. Wang, L. Zhou, L. Qiu, D. Lu, Y. Wu and X.-B. Zhang, *Analyst*, 2015, **140**, 5563-5569.

6 C. Rivas, G. J. Stasiuk, J. Gallo, F. Minuzzi, G. A. Rutter and N. J. Long, *Inorg. Chem.*, 2013, **52**, 14284-14293.
